# Insulin-Like Growth Factor I (IGF-I) Expressed from an AAV1 Vector Leads to a Complete Reversion of Liver Cirrhosis in Rats

**DOI:** 10.1371/journal.pone.0162955

**Published:** 2016-09-22

**Authors:** Luciano Sobrevals, Mónica Enguita, Jorge Quiroga, Jesús Prieto, Puri Fortes

**Affiliations:** 1 Department of Gene Therapy and Hepatology, Center for Applied Medical Research (CIMA), University of Navarra, Pamplona, Spain; 2 University of Navarra Clinic (CUN), Pamplona, Spain; 3 Centro de Investigación Biomédica en Red de Enfermedades Hepáticas y Digestivas (Ciberehd), IdiSNA, Navarra Institute for Health Research, University of Navarra, Pamplona, Spain; University of Pittsburgh School of Medicine, UNITED STATES

## Abstract

IGF-I modulates liver tissue homeostasis. It is produced by hepatocytes and signals within the liver through IGF-I receptor expressed on hepatic stellate cells (HSCs). Liver cirrhosis is characterized by marked IGF-I deficiency. Here we compared the effect of two different gene therapy vectors encoding IGF-I as a potential treatment for cirrhotic patients. Rats with carbon tetrachloride-induced liver cirrhosis were treated with controls or with adeno-associated virus 1 (AAV) or simian virus 40 (SV40) vectors expressing IGF-I (AAVIGF-I or SVIGF-I) and molecular and histological studies were performed at 4 days, 8 weeks and 16 weeks. Increased levels of IGF-I were observed in the liver as soon as 4 days after vector administration. Control cirrhotic rats showed increased hepatic expression of pro-inflammatory and pro-fibrogenic factors including transforming growth factor beta (TGFβ), tumor necrosis factor-alpha (TNFα), connective tissue growth factor (CTGF), and vascular endothelial growth factor (VEGF) together with upregulation of α-smooth muscle actin (αSMA), a marker of HSC activation. In IGF-I-treated rats the levels of all these molecules were similar to those of healthy controls by week 8 post-therapy. Of note, the decline of TGFβ, CTGF, VEGF and αSMA expression was more rapid in AAVIGF-I treated animals reaching statistical significance by day 4 post-therapy. IGF-I-treated rats showed similar improvement of liver function tests in parallel with upregulation of hepatocyte nuclear factor 4α (HNF4α), a factor that promotes hepatocellular differentiation. A significant decrease of liver fibrosis, accompanied by upregulation of the hepatoprotective and anti-fibrogenic hepatocyte growth factor (HGF), occurred in all IGF-I-treated rats but complete reversal of liver cirrhosis took place only in AAVIGF-I group. Therefore, AAVIGF-I reverts liver cirrhosis in rats, a capability which deserves clinical testing.

## Introduction

Liver cirrhosis results from persisting hepatocellular damage of diverse etiologies. It is characterized by an alteration of organ architecture consisting of the presence of regenerative nodules and surrounding fibrous tissue. Hepatic cirrhosis is the final result of a profound disturbance of liver tissue homeostasis. The liver is a complex ecosystem composed of parenchymal and non-parenchymal cells (including hepatic stellate cells–HSC-, Kupffer cells, endothelial cells and lymphocytes) that communicate through a fluent cross-talk of biochemical signals [1]. Continuing hepatocyte injury determines persisting liver cell regeneration. The transformation of the quiescent hepatocyte into a replicative cell leads to downregulation of nuclear factors, such as HNF4α, that govern the mature hepatocyte phenotype with resulting decrease of hepatocellular function. On the other hand, liver cell injury induces pro-inflammatory and pro-fibrogenic factors, such as TNFα, TGFβ, platelet-derived growth factor (PDGF) and VEGF, that promote HSC activation leading to αSMA upregulation and increased collagen synthesis [2]. In advanced liver cirrhosis hepatocyte dedifferentiation [3,4] and increased collagen deposition lead to hepatic insufficiency and portal hypertension, two pathophysiological features responsible for the appearance of complications such as bleeding from esophageal varices, ascites and encephalopathy. At this stage of the disease liver transplantation is the only available curative therapy but this option can be offered to only a limited proportion of patients due to organ shortage and the frequent occurrence of contraindications resulting from co-morbidities or old age. Moreover, in transplanted patients maintained immunosuppression is associated with increased oncogenic risk and infections [5,6] and represents a significant economic burden. Clearly, alternative therapies for advanced liver cirrhosis are much needed.

IGF-I is a typical product of the differentiated hepatocyte [7] and in fact most of circulating IGF-I is of hepatic origin. IGF-I deficiency occurs from the early stages of liver cirrhosis and serum IGF-I may become undetectable in advanced disease [8,9]. IGF-I displays trophic functions systemically acting on intestine, muscle, bone, cartilage and gonads, but it also modulates liver tissue homeostasis [10]. Although *in vitro* studies have shown that IGF-I may stimulate collagen synthesis by isolated HSC, *in vivo* data indicate that IGF-I mediates cytoprotective and anti-fibrogenic effects within the liver. In a transgenic mouse model expressing IGF-I under the control of αSMA promoter it has been shown that IGF-I induces HSC to release HGF which is a potent hepatoprotective and anti-fibrogenic factor [10]. Moreover in rats with experimental cirrhosis, low doses of recombinant IGF-I resulted in a significant reduction of liver fibrosis accompanied by improvement of liver function [11,12]. Besides, mesenchymal stromal cells engineered to produce IGF-I significantly decrease liver fibrosis in mice [13]. Thus, in liver cirrhosis IGF-I deficiency may likely impair the expression of hepatoprotective factors facilitating HSC activation and collagen production.

Based on this notion we have previously used an SV40-based vector to transduce the cirrhotic liver in order to restore intrahepatic expression of IGF-I [14]. We observed that the liver of rats treated with SV40 vector encoding IGF-I (SVIGF-I) showed significant reduction of fibrogenesis and increased fibrolysis together with increased expression of HGF and HNF4α and improved hepatocellular function. Although SVIGF-I was able to induce partial cirrhosis regression, this vehicle is not liable to clinical translation due to uncertainties regarding safety and technical limitations to produce the amount of vector needed for human application. Unlike SV40 vectors, AAV-based gene therapy has been widely and safely used in a diversity of clinical trials [15,16]. Thus, in the present study we have explored the effect of an AAV vector expressing IGF-I (AAVIGF-I), as compared to SVIGF-I, in the treatment of experimental cirrhosis. Here we show that in the liver of cirrhotic rats treated with AAVIGF-I there was a rapid de-activation of HSC in association with downregulation of pro-inflammatory and pro-fibrogenic factors and upregulation of HGF, HNF4α and metalloprotease activity leading to fibrosis regression and improvement of liver function. Unexpectedly, complete reversal of liver cirrhosis was only observed in AAVIGF-I treated rats. Our present findings provide the basis for clinical testing of AAVIGF-I in advanced liver cirrhosis.

## Materials and Methods

### Animal model of established liver cirrhosis

This study was performed following the regulations of the Animal Care Ethical Committee from the University of Navarra. The protocol was approved by the Committee (protocol 037–10) and by a special agent of the Government of Navarra. All surgery was performed under sodium pentobarbital anaesthesia and all possible efforts were made to ensure the wellbeing of the animals and minimize their suffering. Cirrhosis was induced in male Sprague-Dawley rats of (180–200 g), with weekly intragastric administrations of carbon tetrachloride (CCl_4_, Riedel-de Haën) for 8 weeks, as described and 400mg/l of phenobarbital in the drinking water [14]. In brief, the initial CCl_4_ dose was 20 μl per rat. Subsequent doses were adjusted based on the change in body weight 48 hours after the last dose. Normal weight loss ranged from 5–15%. Animals with severe weight loss (higher than 20%), poor motility, difficult access to food or water or lack of response to external stimuli were sacrificed in a CO_2_ chamber. All rats were observed at least twice daily until death. Following this protocol, ascites was apparent in some of the animals. Thirty percent of the animals died during cirrhosis induction and one percent during surgery. Blood samples were collected from the retro-orbital plexus and serum markers were analyzed as a way to evaluate progression of established cirrhosis.

### Construction of pAAVIGF-I

Plasmid pAAV-GFP and pSV-IGF-I [17] were digested with Bgl II and Xho I and ligated to replace the CMV-GFP cassette by the rat IGF-I sequence. The resulting plasmid was digested with Bgl II and a blunt-end ligation was performed to clone in the Bgl II site the liver specific promoter a1antitrypsin (a1AT) fused to the enhancer of albumin (Ealb). The enhancer-promoter sequence was obtained after digestion of pEalba1ATLuc with Xho I and Hind III [18]. In the context of AAV1 vectors, this liver specific promoter drives expression only in rat liver, preferentially in hepatocytes compared to HSCs, Kupffer and endothelial cells [19].

### Inoculation of vectors and experimental protocol

**Fig 1:**
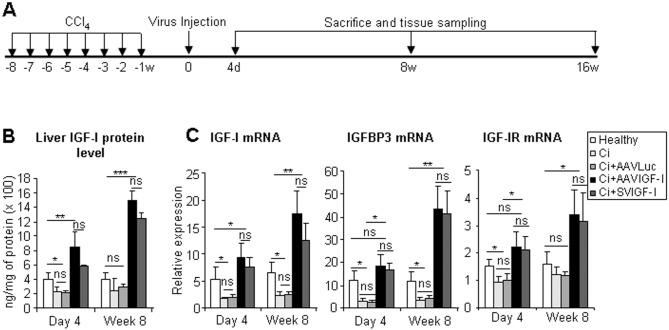
Functional IGF-I is expressed in the liver after administration of AAVIGF-I or SVIGF-I. (a) Schematic of the experimental protocol used. Healthy animals were untreated, or used to induce liver cirrhosis by intragastric administration of CCl_4_ once a week for eight weeks (w). One week after the end of the cirrhosis induction protocol, cirrhotic animals were treated with saline, AAVLuc, AAVIGF-I or SVIGF-I by intra-arterial administration. Animals were sacrificed 4 days, 8 weeks and 16 weeks after vector inoculation. Healthy animals were sacrificed in parallel as controls. Blood was collected and liver samples were processed for histology, and purification of RNA and proteins for further analysis. (b) Analysis of liver IGF-I expression and activity. Liver samples were obtained from healthy, cirrhotic animals treated with saline (Ci), AAVLuc (Ci+AAVLuc), AAVIGF-I (Ci+AAVIGF-I) or SVIGF-I (Ci+SVIGF-I). Total IGF-I protein (a) and IGF-I, IGFBP3 and IGF-IR mRNAs were quantified by ELISA and qRT-PCR in liver extracts. Transcript levels shown are relative to glyceraldehyde 3-phosphate dehydrogenase (GAPDH) mRNA levels. Samples were obtained 4 days or 8 weeks after vector inoculation. Error bars denote standard deviations. Significant and non-significant (ns) differences are highlighted.

Recombinant double stranded AAV vectors encoding IGF-I were produced in packaging cells as described [19]. The inverted terminal repeats of AAV2 were used and the virus was pseudotyped with AAV1. AAV1 was chosen because this serotype transduces rat liver efficiently [20]. The vector that expresses IGF-I was named AAVIGF-I and has the liver specific promoter a1antitrypsin fused to the enhancer of albumin (a1AT). AAVLuc [19] and SVLuc, both expressing luciferase (Luc), were used as negative controls. SVIGF-I expresses IGF-I from a SV40 vector and was used as a positive control [17]. For evaluation of AAVIGF-I (Fig 1a) cirrhotic animals were injected in the hepatic artery with 200 μl of saline (n = 13), AAVLuc (n = 14), AAVIGF-I (n = 17) or SVIGF-I (n = 10). 3.4 x 10^9^ viral particles (vp) of AAVLuc or AAVIGF-I were injected per animal. Regarding SVIGF-I the dose of 10^11^ vp/rat was used as it was previously shown to prevent and partially revert liver cirrhosis in rats [14,17]. A group of healthy rats of the same age (n = 10) was used as an additional control. Luciferase expression was analyzed in AAVLuc treated animals once a week during all the experimental protocol to ensure proper transgene expression. To analyze early and medium-term molecular changes occurring in the liver of cirrhotic rats treated with SVIGF-I or AAVIGF-I animals were sacrificed and blood samples were collected at 4 days (Healthy = 5, saline = 4, AAVLuc = 4, AAVIGF-I = 6, SVIGF-I = 3) and 8 weeks (Healthy = 5, saline = 4, AAVLuc = 5, AAVIGF-I = 5, SVIGF-I = 3) after vector administration (Fig 1a). The long-term effect of the two IGF-I expressing vectors on cirrhosis regression was assessed by studying liver histology at four months in cirrhotic rats treated with saline (n = 5), AAVLuc (n = 5), AAVIGF-I (n = 6) or SVIGF-I (n = 4).

### Serum biochemistry

Serum transaminases (alanine (ALT) and aspartate aminotransferase (AST) and alkaline phosphate (ALP)), albumin and bilirubin, were measured (ABX diagnostics) in a Hitachi autoanalyzer (Roche).

### Liver histology and immunohistochemistry

Liver collagen content was assessed by Sirius red staining and scored by imaging analysis (AnalySIS 3.1, Soft Imaging System) as described [14]. αSMA was stained with a mouse monoclonal antibody (1A4, Sigma) diluted 1:400.

### Proteins and RNA analysis

Total liver IGF-I (OCTEIA Rat/mouse IGF-I, Vitro), MMP-2 (Matrix Metalloproteinase-2 Activity Assay Biotrak System) and MMP-9 (Matrix Metalloproteinase-9 Activity Assay Biotrak System) were measured in liver extracts by ELISA. MMP activity was also evaluated in liver extracts with a fluorigenic peptide (Mca-Pro-Leu-Gly-Leu-Dpa-Ala-Arg-NH_2;_ R&D system) [21]. Recombinant human MMP-2 (Calbiochem) was used as positive control. TGFβ, TNFα, interleukin 6 (IL-6) (all of them from Elisa BD OptE1A^™^, BD biosciences) and HGF (Institute of immunology Co, Ldt) were measured in liver extracts. Total RNA was extracted as described previously [17]. Quantitative RT-PCRs (qRT-PCRs) were done using the primers and conditions listed in S1 Table and described in [22].

### Statistical analysis

Data are expressed as means ± standard deviation. The normality of quantitative variables was assessed with D´Agostino and Pearson omnibus normality test. Data were compared with analysis of variance (ANOVA) and multiple Bonferroni-corrected Mann–Whitney U-tests were used as post hoc comparisons. When the normality could not be proven, Kruskal–Wallis tests were used to evaluate global differences and Dunns was used as post-test. Control groups that were proven undistinguishable were pooled for increased sensitivity. Equivalence was calculated with two one-sided test (TOST) [23]. Samples were considered equivalent when the range of inferential confidence interval with a probability of 95% (ICI_95_) was contained within the range of equivalence defined as three standard deviations of the mean of the healthy sample. Differences were deemed significant for a real alpha of 0.05. * denotes p≤0.05, ** p≤0.01 and *** p≤0.001. All statistical analyses were carried out with SPSS v11.0 (SPSS Inc).

## Results

### AAVIGF-I restores hepatic IGF-I expression in liver cirrhosis

Different groups of cirrhotic rats were treated with either saline (Ci), AAVLuc, AAVIGF-I or SVIGF-I one week after completion of the cirrhosis induction protocol and a group of healthy rats were used as normal controls. Animals were sacrificed and analyzed for different parameters at day 4 and week 8 post-therapy and some rats were sacrificed at week 16 to assess the long term effect of the therapy on liver histology (Fig 1a).

A significant decrease of IGF-I expression was observed in control cirrhotic livers (Ci and AAVLuc) in comparison to healthy rats and this was accompanied by reduced hepatic expression of the classical IGF-I target gene IGF binding protein 3 (IGFBP3) (Fig 1b and 1c). Eight weeks after therapy, a significant rise in the hepatic levels of IGF-I mRNA and protein was observed in both SVIGF-I and AAVIGF-I groups, reaching values above normal levels. This was associated with a parallel elevation of IGFBP3. A concomitant increase in serum levels of IGF-I was not observed (data not shown). The enhancement of liver IGF-I expression could be detected as soon as day 4 in AAVIGF-I and SVIGF-I treated rats (Fig 1b). As indicated above while IGF-I is a product of hepatocytes, IGF-I receptor (IGF-IR) is mainly expressed by HSC [19]. Interestingly, IGF-IR was upregulated in all animals that received IGF-I encoding vectors (Fig 1c), indicating that restoration of liver IGF-I levels causes increased sensitivity to the intrahepatic effects of this hormone.

### AAVIGF-I suppresses intrahepatic expression of pro-inflammatory and pro-fibrogenic factors and induces rapid HSC deactivation

**Fig 2:**
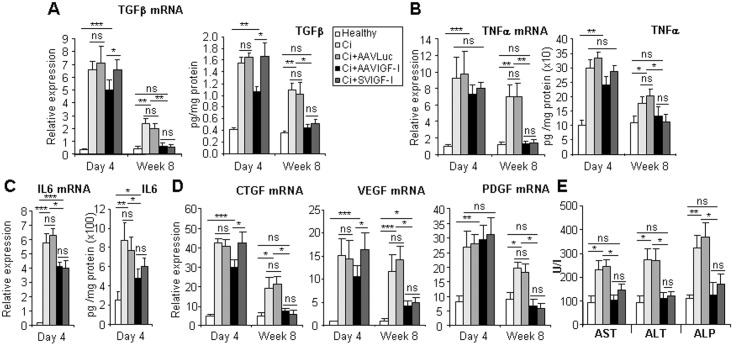
Analysis of liver damage, pro-inflammatory and pro-fibrogenic factors. Animals were treated as described in Fig 1 and TGFβ (a), TNFα (b) and IL-6 (c) mRNA and protein levels were evaluated in liver extracts by qRT-PCR or ELISA. CTGF, VEGF and PDGF mRNA levels were also measured. Transcript levels shown are relative to GAPDH mRNA levels. (e) Transaminases AST, ALT and ALP were quantified in the serum of the animals 8 weeks after vector administration. Error bars denote standard deviations. Significant and non-significant (ns) differences are highlighted.

Both AAVIGF-I and SVIGF-I induced a significant reduction in the intrahepatic expression of pro-inflammatory and pro-fibrogenic factors including IL-6, TNFα, TGFβ, CTGF, VEGF and PDGF (Fig 2). Equivalence tests indicated that at week 8 after therapy with the exception of VEGF and IL-6, which was not detected, the values of all these molecules were similar in healthy livers and in animals treated with either AAVIGF-I or SVIGF-I (Fig 2). However, they remained significantly above normal levels in both Ci and AAVLuc groups. A similar result was observed when evaluating the levels of liver enzymes (Fig 2e). Notably, we observed a rapid and significant decline in the expression of TGFβ (both mRNA and protein), CTGF and VEGF by day 4 after therapy in AAVIGF-I treated rats but not in those given SVIGF-I (Fig 2a and 2d).

**Fig 3:**
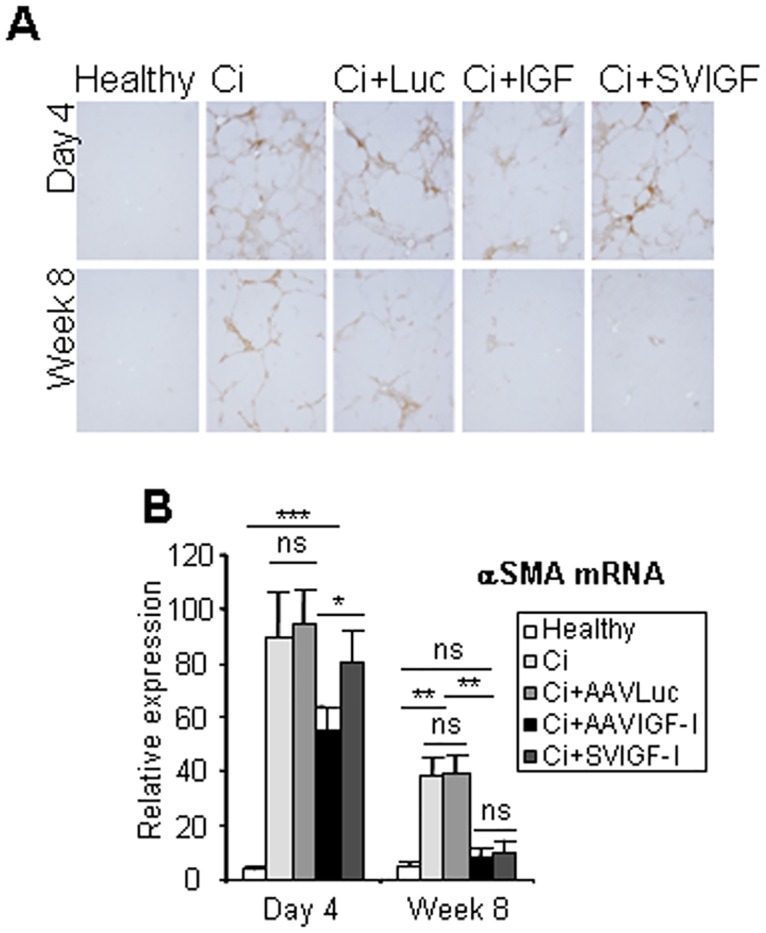
Analysis of HSCs. Animals were treated as described in Fig 1 and αSMA expression and localization was evaluated by immunohistochemistry (a) and qRT-PCR (b). Transcript levels shown are relative to GAPDH mRNA levels. Error bars denote standard deviations. Significant and non-significant (ns) differences are highlighted.

Consonant with these findings, immunohistochemical and qRT-PCR analyses showed that hepatic expression αSMA, a marker of HSC activation, was not statistically different from healthy values by week 8 in both AAVIGF-I and SVIGF-I treated groups while it remained significantly elevated in Ci and AAVLuc animals. Interestingly, at day 4, αSMA was found to be significantly downregulated in AAVIGF-I treated rats but not in those given SVIGF-I, indicating a more rapid effect of the former vector in the induction of HSC deactivation (Fig 3).

### AAVIGF-I upregulates HGF and enhances the expression and activity of metalloproteases (MMPs) in liver tissue

**Fig 4:**
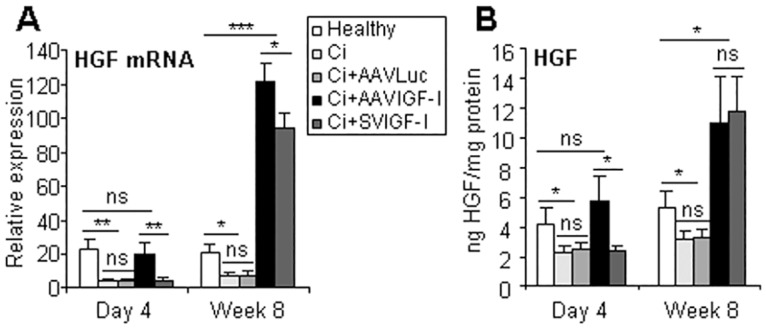
Analysis of HGF. Animals were treated as described in Fig 1 and HGF mRNA (a) and protein (b) levels were evaluated by qRT-PCR or ELISA in liver extracts. Transcript levels shown are relative to GAPDH mRNA levels. Error bars denote standard deviations. Significant and non-significant (ns) differences are highlighted.

Hepatocyte growth factor (HGF) is produced in the liver by non-parenchymal cells and acts as a potent cytoprotective and anti-fibrogenic factor [24]. One important effect of HGF is the induction of MMPs which are involved in degradation of extracellular matrix [24]. It has been shown that intrahepatic expression of IGF-I stimulates HGF production by HSC [10,19]. Confirming these data we found that livers from cirrhotic rats treated with either AAVIGF-I or SVIGF-I showed increased hepatic HGF mRNA and protein levels at week 8 after therapy and that HGF upregulation could already been observed at day 4 in the former group but not in the later (Fig 4). Again these results indicate a quicker effect of AAVIGF-I as compared to SVIGF-I in the modulation of anti-fibrogenic factors.

**Fig 5:**
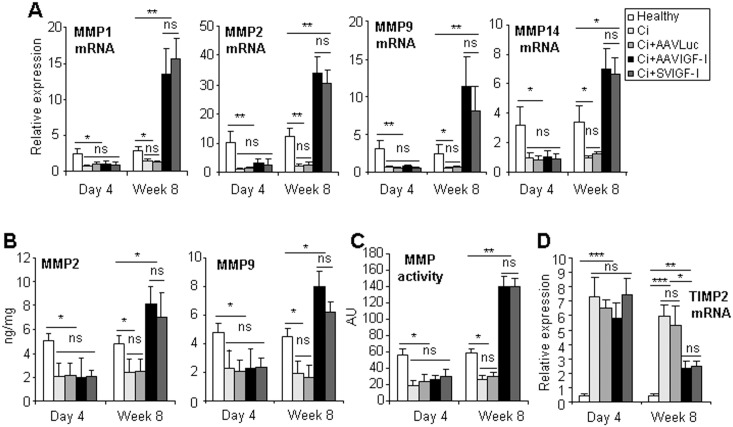
Analysis of MMPs and MMP inhibitors. Animals were treated as described in Fig 1 and MMP-1, 2, 9 and 14 mRNAs (a) and MMP-2 and 9 proteins (b) were evaluated in liver extracts by qRT-PCR or ELISA respectively. Total MMP activity in liver extracts (c) and the levels of MMP inhibitor TIMP-2 mRNA (d) were also evaluated. Transcript levels shown are relative to GAPDH mRNA levels. Error bars denote standard deviations. Significant and non-significant (ns) differences are highlighted.

In agreement with HGF upregulation, we observed a significant elevation of MMP activity, MMP-1, MMP-2, MMP-9 and MMP-14 mRNA, and of MMP-2 and MMP-9 protein in the livers of cirrhotic rats treated with either AAVIGF-I or SVIGF-I (Fig 5). These changes were accompanied by a significant reduction in the expression of tissue inhibitor of metalloproteinases-2 (TIMP-2), a potent inhibitor of MMPs. The effect of the therapy on MMPs and TIMP-2 was apparent at week 8 but not at day 4 after vector injection.

### Treatment of cirrhotic rats with AAVIGF-I leads to complete cirrhosis reversion with disappearance of liver fibrosis and improvement of hepatocellular function

**Fig 6:**
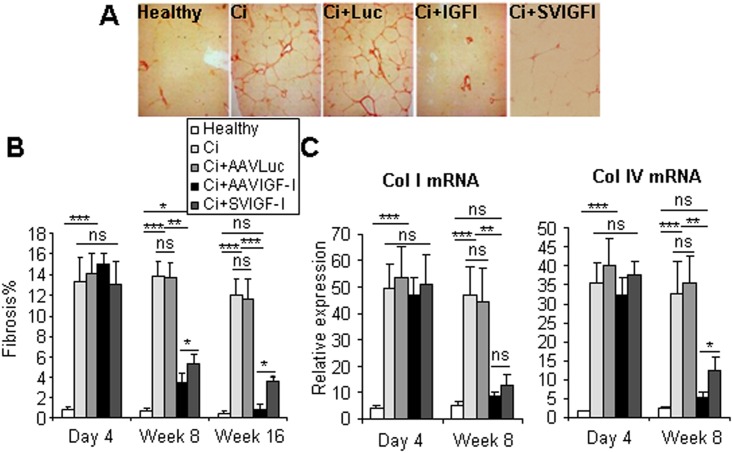
Analysis of liver fibrosis. Animals were treated as described in Fig 1 and liver fibrosis was evaluated by quantification (b) of Sirius red staining 16 weeks after vector administration (a) or by qRT-PCR of collagen I and collagen IV mRNA performed in samples collected at the indicated times (c). Collagen mRNA levels shown are relative to GAPDH mRNA levels. Error bars denote standard deviations. Significant and non-significant (ns) differences are highlighted.

A central finding in this study was that liver fibrosis was not statistically different in healthy animals and rats analyzed at 16 weeks after therapy with AAVIGF-I (Fig 6). Of note, collagen deposition was not completely normalized in rats which received SVIGF-I, although they exhibited a significant reduction of fibrosis score. Similar results were obtained when collagen IV expression was evaluated. While collagen I levels were not statistically different in AAVIGF-I and SVIGF-I-treated rats, collagen IV levels were significantly increased in SVIGF-I compared to AAVIGF-I-treated rats. Interestingly, the levels of collagen I and IV in animals treated with AAVIGF-I for 8 weeks were not statistically different from those observed in healthy animals.

**Fig 7:**
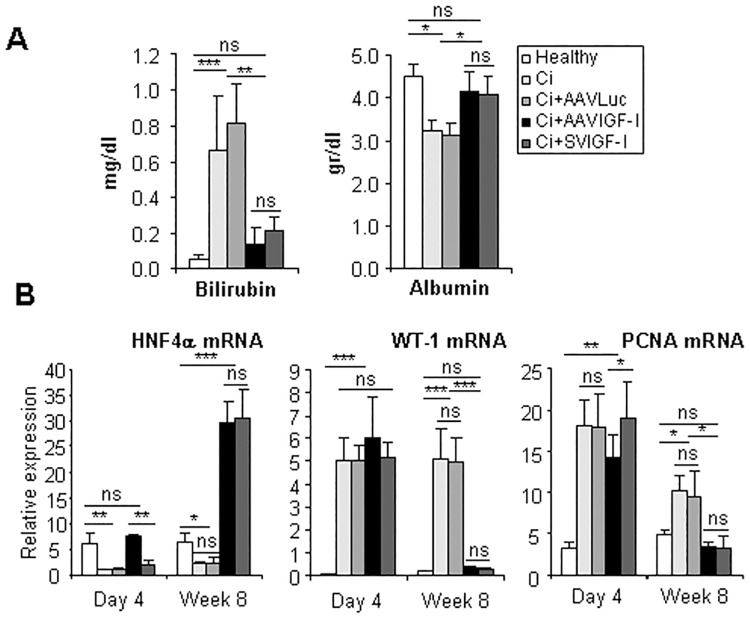
IGF-I treatment restores liver functionality and hepatocyte differentiation. (a) Bilirubin and albumin were quantified in the serum of animals treated as described in Fig 1, 8 weeks after vector administration. (b) mRNA levels of the maturation factor HNF4α, the differentiation factor WT-1 and the proliferation factor PCNA were evaluated by qRT-PCR in liver extracts of animals described in Fig 1. Transcript levels shown are relative to GAPDH mRNA levels. Error bars denote standard deviations. Significant and non-significant (ns) differences are highlighted.

Of remark, these beneficial effects on hepatic fibrosis were accompanied in both AAVIGF-I and SVIGF-I groups by a general normalization of hepatocellular function, as estimated by serum bilirubin (non-significantly different between healthy and IGF-I-treated animals) and albumin levels (which were similar in healthy and IGF-I-treated animals) observed at 8 weeks post-therapy (Fig 7). Improvement of liver function was associated with upregulation of the hepatocyte differentiating factor HNF4α and downregulation of Wilms' tumor 1 (WT-1), a nuclear factor upregulated in liver cirrhosis which has been shown to mediate hepatocellular dedifferentiation (Fig 7). Interestingly, similar to other molecules like HGF and TGFβ, the effect on HNF4α occurred more rapidly in the AAVIGF-I group. Hepatic proliferating cell nuclear antigen (PCNA) expression, a surrogate of liver cell proliferation, showed a significant reduction at day 4 in AAVIGF-I treated rats being the levels comparable to those present in healthy livers at week 8 after therapy in both AAVIGF-I and SVIGF-I groups. This finding likely reflects the quiescence of the mature hepatocyte and the hepatocyte differentiating effect of IGF-I encoding vectors.

## Discussion

Although liver transplantation has represented a formidable advance in the management of liver cirrhosis, this therapeutic approach has important limitations. These include organ shortage, frequent occurrence of contraindications, procedure-associated mortality and morbidity, the need for long-term immunosuppression with increased risk of infections and tumor development and high cost [25]. Consequently, translational research aimed at testing novel therapies for patients with decompensated liver cirrhosis is an urgent medical task.

Liver cirrhosis has long been considered an irreversible condition. However, recent evidence in persistently abstinent alcoholics, in patients with hepatitis B virus-induced cirrhosis treated for years with anti-polymerase drugs and in patients with hepatitis C virus (HCV)-induced cirrhosis who cleared the virus after antiviral therapy, indicates that liver cirrhosis can revert partially or completely [26–29]. Clinical data showing that the cirrhotic liver possesses tissue plasticity indicate that factors implicated in liver tissue homeostasis can effectively achieve organ remodeling upon cessation of the injurious agent.

Within the liver ecosystem, IGF-I represents a signal emanating from differentiated hepatocytes which modulates the functionality of non-parenchymal cells. These, in turn, secrete mediators affecting hepatocyte survival, differentiation and function [10,24,30–32]. In liver cirrhosis hepatocellular insufficiency causes marked IGF-I deficiency [8,33] and this situation could benefit from hormone replacement therapy. In fact a pilot randomized double-bind clinical trial testing recombinant IGF-I given subcutaneously for 4 months to cirrhotic patients demonstrated amelioration of liver biosynthetic functions with significant improvement of Child-Pugh score [34]. Parenteral administration of IGF-I protein induces both intrahepatic and systemic hormonal effects. However, when looking for a preferential effect of IGF-I within the liver, as when this hormone is intended for remodeling the cirrhotic tissue, a gene therapy approach is desirable. Targeting the liver with a hepatotropic gene therapy vector would enable transgenic IGF-I to be orthotopically synthesized by hepatocytes in close vicinity to non-parenchymal liver cells located in the fibrous scar where IGF-IR is expressed [14]. In this way, only a moderate rise of intrahepatic IGF-I levels, which per se do not elevate serum IGF-I concentration (data not shown), is enough to trigger a signaling cascade within the cirrhotic liver leading to regression of histopathological and functional alterations. According to these concepts, it has been shown that gene therapy of experimental liver cirrhosis in rats using an SV40 based vector caused attenuation of inflammation, partial fibrosis regression and improved hepatocellular function [14]. This study, however, was performed with a vector which is not amenable to clinical use. In order to provide support for clinical translation of IGF-I based gene therapy of liver cirrhosis, in the present work we have used an AAV vector which has been widely and safely employed in diversity of liver-directed gene therapy clinical trials.

Here we show that AAVIGF-I is a potent tool acting within the liver to re-route hepatic tissue homeostasis. With the vector doses used in this study we found that, in comparison to control cirrhotic rats, the levels of intrahepatic IGF-I were increased at day 4 and week 8 in both AAVIGF-I and SVIGF-I groups. We observed that, unlike SVIGF-I, AAVIGF-I displayed its effects very quickly, being detectable as soon as 4 days after vector injection. At this early time point, a significant reduction of αSMA expression together with downregulation of the profibrogenic molecules TGFβ, CTGF and VEGF and upregulation of HGF and HNF4α was present in AAVIGF-I treated animals but not in those which received SVIGF-I. Differences in the therapeutic effects between the two vectors were also observed at week 8. At this moment, the AAVIGF-I group showed normal collagen IV mRNA values while they were still elevated in the SVIGF-I group. Remarkably, variances between the two vectors were also apparent at week 16, on which SVIGF-I cirrhotic rats still showed significant liver fibrosis while liver histology reverted to normal in animals treated with AAVIGF-I. Furthermore, similar liver fibrosis was observed in animals treated with SVIGF-I for 16 or 25 weeks (data not shown). We cannot exclude the possibility that the different outcome results from a faster and/or greater expression of IGF-I from AAVIGF-I than from SVIGF-I. However, the statistical analysis shows that the levels of IGF-I observed 4 days and 8 weeks after administration of SVIGF-I and AAVIGF-I are not significantly different. Therefore, we hypothesize that the different therapeutic efficacy observed with AAVIGF-I and SVIGF-I may result from the different targeting of these vectors into the liver. While both SVIGF-I and AAVIGF-I target preferentially hepatocytes, a significant expression of IGF-I can be observed in HSCs, Kupffer and endothelial cells in livers treated with SVIGF-I, which employs a ubiquitous SV40 promoter for transgene expression [14]. However, very little transgene expression is observed in non-parenchymal cells when the AAV1 with the liver specific promoter is used [19].

In all cirrhotic animals treated with IGF-I expressing vectors regression of hepatic fibrosis was associated with dampening of fibrogenic mediators, enhanced expression of MMPs and downregulation of TIMP-2. We believe that these factors played a major role in the resolution of liver fibrosis. In addition AAVIGF-I, like SVIGF-I, also proved to be highly effective at inhibiting inflammation, attenuating liver damage and stimulating hepatocyte differentiation and hepatocellular function. These effects, likely mediated in part by HGF upregulation, favor hepatocellular quiescence as shown by lower PCNA levels in IGF-I treated livers.

A concern for the translation of AAVIGF-I therapy into the clinic is that IGF-I is a growth factor which participates in the development of different malignancies [35]. Liver cirrhosis is a preneoplastic condition which predisposes to hepatocellular carcinoma (HCC), a tumor where IGF-IR activation has been shown to promote tumor growth [9]. However, in the cirrhotic liver HCC develops in a tissue with very low IGF-I expression [36]. HCC oncogenesis is associated with strong upregulation of insulin growth factor 2 (IGF-II); this molecule, and not IGF-I, appears to be the oncogenic IGF-IR ligand in HCC [9]. In fact, it has been shown that post-resection recurrence of HCC is associated with the expression in the cirrhotic non-tumor tissue of genes reflecting hepatocellular dedifferentiation and poor liver function while good prognosis after resection is linked to preserved IGF-I expression [37]. Here we found that AAVIGF-I improves liver function and upregulates HNF4α which promotes hepatocyte differentiation while downregulates WT-1 which incites hepatocellular dedifferentiation and HCC development [3,38]. On the other hand as TNFα and IL-6 signaling have been also shown to be involved in HCC oncogenesis [37], the observed anti-inflammatory effects of IGF-I encoding vectors might oppose tumor development in the cirrhotic liver. Thus, there are grounds to think that AAVIGF-I therapy in addition to reducing fibrosis and improving hepatocellular function might also decrease the oncogenic risk in liver cirrhosis. In this regard it should be mentioned that we have performed necropsy studies one year after the administration of 3.4 x 10^9^ vp/rat of AAVIGF-I into cirrhotic animals and no tumors were found and non-significant differences were observed after analysis of several parameters in blood and urine and histopathological analyses of several organs from 3 healthy animals and cirrhotic animals injected with saline (n = 4), AAVLuc (n = 2) or AAVIGF-I (n = 4) (data not shown). And the same result was observed in necropsy analyses performed one year after the administration of 10^12^ vp/rat of AAVIGF-I. In this study, non-significant differences were found after analysis of several parameters in blood and histopathological analyses of several organs from 4 healthy animals and cirrhotic animals injected with saline (n = 4), AAVLuc (n = 4) or AAVIGF-I (n = 6) (data not shown). Moreover, mice inoculated with AAVIGF-I before or 6 months after treatment with diethylnitrosamine (DEN), which induces hepatocarcinogenesis in mice, did not show more tumors or larger tumors than control animals (data not shown). While these results suggest that treatment with AAVIGF-I is safe, unpredicted unwanted secondary effects derived from long-term expression of exogenous IGF-I in the liver could be avoided by using inducible promoters. These promoters should be switched-off once the therapeutic effect has been achieved. Further, it should be stressed that clinical trials should be performed in cirrhotic patients without detectable tumors.

Other issues should be stablished before the beginning of the clinical trial. We have used AAV1 vectors in our studies because these vectors transduce rat liver efficiently [20]. However, this serotype should not be used in patients, as human liver transduction by this vector has not been studied and both the prevalence of serum anti-AAV1 IgG and the neutralizing factor seroprevalence is high. Instead we recommend the use of a vector whose tropism for human liver has been well-documented, such as AAV8 [39]. Moreover, initial clinical trial should better include patients whose underlying cause of liver cirrhosis has been eliminated, such as patients who have cleared HCV after antiviral therapy. Notwithstanding, IGF-I might also demonstrate therapeutic efficacy in patients with active liver cirrhosis. In fact, previous results have shown that SVIGF-I treatment decreases liver cirrhosis progression in rat liver [34] and significant improvement in liver function has been observed in patients with alcoholic cirrhosis treated with recombinant IGF-I for 4 months [34].

In conclusion, IGF-I based gene therapy using an AAV vector promotes reversal of experimental liver cirrhosis. These findings provide support for translational studies in cirrhotic patients with advanced disease.

## Supporting Information

S1 Table Conditions and primers used in the qRT-PCRs showed in this work. (XLSX)

## References

[1] Blouin A, Bolender RP, Weibel ER. Distribution of organelles and membranes between hepatocytes and nonhepatocytes in the rat liver parenchyma. A stereological study. J Cell Biol. 1977;72: 441–455. 833203 10.1083/jcb.72.2.441 PMC2110997

[2] Friedman SL. Mechanisms of hepatic fibrogenesis. Gastroenterology. 2008;134: 1655–1669. 10.1053/j.gastro.2008.03.003 18471545 PMC2888539

[3] Berasain C, Herrero JI, Garcia-Trevijano ER, Avila MA, Esteban JI, Mato JM, et al Expression of Wilms' tumor suppressor in the liver with cirrhosis: relation to hepatocyte nuclear factor 4 and hepatocellular function. Hepatology. 2003;38: 148–157. 12829997 10.1053/jhep.2003.50269

[4] Elizalde M, Urtasun R, Azkona M, Latasa MU, Goni S, Garcia-Irigoyen O, et al Splicing regulator SLU7 is essential for maintaining liver homeostasis. J Clin Invest. 2014;124: 2909–2920. 10.1172/JCI74382 24865429 PMC4071377

[5] Rodriguez-Peralvarez M, De la Mata M, Burroughs AK. Liver transplantation: immunosuppression and oncology. Curr Opin Organ Transplant. 2014;19: 253–260. 10.1097/MOT.0000000000000069 24685671 PMC4025587

[6] Issa DH, Alkhouri N. Long-term management of liver transplant recipients: A review for the internist. Cleve Clin J Med. 2015;82: 361–372. 10.3949/ccjm.82a.14072 26086495

[7] Scott CD, Martin JL, Baxter RC. Rat hepatocyte insulin-like growth factor I and binding protein: effect of growth hormone in vitro and in vivo. Endocrinology. 1985;116: 1102–1107. 2982573 10.1210/endo-116-3-1102

[8] Conchillo M, Prieto J, Quiroga J. Insulin-like growth factor I (IGF-I) and liver cirrhosis. Rev Esp Enferm Dig. 2007;99: 156–164. 17516829 10.4321/s1130-01082007000300007

[9] Enguita-German M, Fortes P. Targeting the insulin-like growth factor pathway in hepatocellular carcinoma. World J Hepatol. 2014;6: 716–737. 10.4254/wjh.v6.i10.716 25349643 PMC4209417

[10] Sanz S, Pucilowska JB, Liu S, Rodriguez-Ortigosa CM, Lund PK, Brenner DA, et al Expression of insulin-like growth factor I by activated hepatic stellate cells reduces fibrogenesis and enhances regeneration after liver injury. Gut. 2005;54: 134–141. 15591519 10.1136/gut.2003.024505 PMC1774353

[11] Castilla-Cortazar I, Garcia M, Quiroga J, Diez N, Diez-Caballero F, Calvo A, et al Insulin-like growth factor-I reverts testicular atrophy in rats with advanced cirrhosis. Hepatology. 2000;31: 592–600. 10706548 10.1002/hep.510310308

[12] Lorenzo-Zuniga V, Rodriguez-Ortigosa CM, Bartoli R, Martinez-Chantar ML, Martinez-Peralta L, Pardo A, et al Insulin-like growth factor I improves intestinal barrier function in cirrhotic rats. Gut. 2006;55: 1306–1312. 16434425 10.1136/gut.2005.079988 PMC1860012

[13] Fiore EJ, Bayo JM, Garcia MG, Malvicini M, Lloyd R, Piccioni F, et al Mesenchymal stromal cells engineered to produce IGF-I by recombinant adenovirus ameliorate liver fibrosis in mice. Stem Cells Dev. 2015;24: 791–801. 10.1089/scd.2014.0174 25315017 PMC4356192

[14] Sobrevals L, Rodriguez C, Romero-Trevejo JL, Gondi G, Monreal I, Paneda A, et al Insulin-like growth factor I gene transfer to cirrhotic liver induces fibrolysis and reduces fibrogenesis leading to cirrhosis reversion in rats. Hepatology. 2010;51: 912–921. 10.1002/hep.23412 20198635

[15] Stone D. Novel viral vector systems for gene therapy. Viruses. 2010;2: 1002–1007. 10.3390/v2041002 21994667 PMC3185661

[16] Yla-Herttuala S. Endgame: glybera finally recommended for approval as the first gene therapy drug in the European union. Mol Ther. 2012;20: 1831–1832. 10.1038/mt.2012.194 23023051 PMC3464639

[17] Vera M, Sobrevals L, Zaratiegui M, Martinez L, Palencia B, Rodriguez CM, et al Liver transduction with a simian virus 40 vector encoding insulin-like growth factor I reduces hepatic damage and the development of liver cirrhosis. Gene Ther. 2007;14: 203–210. 17024107 10.1038/sj.gt.3302858

[18] Kramer MG, Barajas M, Razquin N, Berraondo P, Rodrigo M, Wu C, et al In vitro and in vivo comparative study of chimeric liver-specific promoters. Mol Ther. 2003;7: 375–385. 12668133 10.1016/s1525-0016(02)00060-6

[19] Sobrevals L, Enguita M, Rodriguez C, Gonzalez-Rojas J, Alzaguren P, Razquin N, et al AAV vectors transduce hepatocytes in vivo as efficiently in cirrhotic as in healthy rat livers. Gene Ther. 2012;19: 411–417. 10.1038/gt.2011.119 21850051

[20] Seppen J, Bakker C, de Jong B, Kunne C, van den Oever K, Vandenberghe K, et al Adeno-associated virus vector serotypes mediate sustained correction of bilirubin UDP glucuronosyltransferase deficiency in rats. Mol Ther. 2006;13: 1085–1092. 16581301 10.1016/j.ymthe.2006.01.014

[21] Knight CG, Willenbrock F, Murphy G. A novel coumarin-labelled peptide for sensitive continuous assays of the matrix metalloproteinases. FEBS Lett. 1992;296: 263–266. 1537400 10.1016/0014-5793(92)80300-6

[22] Barriocanal M, Carnero E, Segura V, Fortes P. Long Non-Coding RNA BST2/BISPR is Induced by IFN and Regulates the Expression of the Antiviral Factor Tetherin. Front Immunol. 2015;5: 655 10.3389/fimmu.2014.00655 25620967 PMC4288319

[23] Walker E, Nowacki AS. Understanding equivalence and noninferiority testing. J Gen Intern Med. 2011;26: 192–196. 10.1007/s11606-010-1513-8 20857339 PMC3019319

[24] Nakamura T, Sakai K, Nakamura T, Matsumoto K. Hepatocyte growth factor twenty years on: Much more than a growth factor. J Gastroenterol Hepatol. 2011;26 Suppl 1: 188–202. 10.1111/j.1440-1746.2010.06549.x 21199531

[25] Chandok N, Watt KD. Burden of de novo malignancy in the liver transplant recipient. Liver Transpl. 2012;18: 1277–1289. 10.1002/lt.23531 22887956

[26] Aghemo A, Colombo M. Cirrhosis regression in chronic hepatitis C: an old tale with a new ending. Gastroenterology. 2009;136: 1447–1449. 10.1053/j.gastro.2009.02.033 19250651

[27] Brown A, Goodman Z. Hepatitis B-associated fibrosis and fibrosis/cirrhosis regression with nucleoside and nucleotide analogs. Expert Rev Gastroenterol Hepatol. 2012;6: 187–198. 10.1586/egh.12.4 22375524

[28] Ellis EL, Mann DA. Clinical evidence for the regression of liver fibrosis. J Hepatol. 2012;56: 1171–1180. 10.1016/j.jhep.2011.09.024 22245903

[29] Manne V, Akhtar E, Saab S. Cirrhosis regression in patients with viral hepatitis B and C: a systematic review. J Clin Gastroenterol. 2014;48: e76–84. 10.1097/MCG.0000000000000162 24921210

[30] Zinchenko YS, Culberson CR, Coger RN. Contribution of non-parenchymal cells to the performance of micropatterned hepatocytes. Tissue Eng. 2006;12: 2241–1251. 16968164 10.1089/ten.2006.12.2241

[31] Casteleijn E, Kuiper J, Van Rooij HC, Koster JF, Van Berkel TJ. Conditioned media of Kupffer and endothelial liver cells influence protein phosphorylation in parenchymal liver cells. Involvement of prostaglandins. Biochem J. 1988;252: 601–605. 3166374 10.1042/bj2520601 PMC1149185

[32] Bouwens L, De Bleser P, Vanderkerken K, Geerts B, Wisse E. Liver cell heterogeneity: functions of non-parenchymal cells. Enzyme. 1992;46: 155–168. 1289080 10.1159/000468782

[33] Fernandez-Rodriguez CM, Prada I, Andrade A, Moreiras M, Guitian R, Aller R, et al Disturbed synthesis of insulinlike growth factor I and its binding proteins may influence renal function changes in liver cirrhosis. Dig Dis Sci. 2001;46: 1313–1320. 11414310 10.1023/a:1010631800505

[34] Conchillo M, de Knegt RJ, Payeras M, Quiroga J, Sangro B, Herrero JI, et al Insulin-like growth factor I (IGF-I) replacement therapy increases albumin concentration in liver cirrhosis: results of a pilot randomized controlled clinical trial. J Hepatol. 2005;43: 630–636. 16024131 10.1016/j.jhep.2005.03.025

[35] Yu H, Rohan T. Role of the insulin-like growth factor family in cancer development and progression. J Natl Cancer Inst. 2000;92: 1472–1489. 10995803 10.1093/jnci/92.18.1472

[36] Su TS, Liu WY, Han SH, Jansen M, Yang-Fen TL, P'eng FK, et al Transcripts of the insulin-like growth factors I and II in human hepatoma. Cancer Res. 1989;49: 1773–1777. 2466561

[37] Hoshida Y, Villanueva A, Kobayashi M, Peix J, Chiang DY, Camargo A, et al Gene expression in fixed tissues and outcome in hepatocellular carcinoma. N Engl J Med. 2008;359: 1995–2004. 10.1056/NEJMoa0804525 18923165 PMC2963075

[38] Perugorria MJ, Castillo J, Latasa MU, Goni S, Segura V, Sangro B, et al Wilms' tumor 1 gene expression in hepatocellular carcinoma promotes cell dedifferentiation and resistance to chemotherapy. Cancer Res. 2009;69: 1358–1367. 10.1158/0008-5472.CAN-08-2545 19190340

[39] Nathwani AC, Tuddenham EG, Rangarajan S, Rosales C, McIntosh J, Linch DC, et al Adenovirus-associated virus vector-mediated gene transfer in hemophilia B. N Engl J Med. 2011;365: 2357–2365. 10.1056/NEJMoa1108046 22149959 PMC3265081

